# Endothelial Dysfunction as the Common Pathway Linking Obesity, Hypertension and Atherosclerosis

**DOI:** 10.3390/ijms262010096

**Published:** 2025-10-16

**Authors:** Ewelina Młynarska, Kinga Bojdo, Hanna Frankenstein, Katarzyna Krawiranda, Natalia Kustosik, Wiktoria Lisińska, Jacek Rysz, Beata Franczyk

**Affiliations:** 1 Department of Nephrocardiology, Medical University of Lodz, 90-419 Łódź, Poland; 2 Department of Nephrology, Hypertension and Internal Medicine, Medical University of Lodz, 90-549 Łodz, Poland

**Keywords:** endothelial dysfunction, endothelial activation, endothelial nitric oxide synthase, cardiometabolic disease, vascular inflammation, chemokines, adipocytokines, atherosclerosis, diabetes, biomarkers

## Abstract

Endothelial dysfunction plays a central role in the pathogenesis of cardiovascular diseases, driven by a complex interplay of oxidative stress, metabolic imbalances, and adipokine dysregulation. Excessive reactive oxygen species reduce nitric oxide bioavailability by impairing endothelial nitric oxide synthase function, leading to vascular inflammation and impaired vasodilation. Adipose tissue-derived hormones such as leptin, adiponectin, and resistin exert opposing effects on vascular homeostasis, influencing inflammation and oxidative stress in obesity and metabolic syndrome. Dyslipidemia, particularly through oxidized LDL, initiates endothelial injury and foam cell formation, accelerating atherosclerosis. Furthermore, hypertension and obesity exacerbate vascular dysfunction by disrupting the balance between vasodilators and vasoconstrictors, enhancing oxidative stress, and altering perivascular adipose tissue function. These interrelated mechanisms contribute to the progression of atherosclerotic cardiovascular disease and diabetic vascular complications. A deeper understanding of these processes is essential for developing targeted interventions to restore endothelial health and reduce cardiometabolic risk.

## 1. Introduction

Cardiovascular disease is the leading cause of death worldwide. Coronary artery disease (CAD) is the most common and is characterized by the accumulation of lipids and immune cells in the subendothelial space of the coronary arteries or atherosclerosis. This process involves the inflammatory response of the vascular endothelium [1].

The endothelium is a single-cell layer lining the interior surface of blood vessels and plays a crucial role in maintaining vascular homeostasis through various protective mechanisms. These include the regulation of vascular tone, permeability, and the modulation of anti-inflammatory, antioxidant, anti-proliferative, and anti-thrombotic pathways. It achieves this by releasing a range of autocrine, paracrine, and endocrine mediators, such as nitric oxide (NO), C-type natriuretic peptide, prostacyclin, and endothelium-derived hyperpolarizing factors [2]. These substances contribute to the suppression of smooth muscle cell proliferation and migration, reduce platelet adhesion and aggregation, and regulate processes involved in thrombosis development [3,4].

Endothelial dysfunction arises when the balance between NO synthesis and degradation is disrupted, leading to the diminished availability of NO. This pathological shift promotes the activation and adhesion of platelets and leukocytes, as well as the release of proinflammatory cytokines. These events enhance vascular permeability to oxidized lipoproteins and inflammatory mediators, ultimately causing structural alterations in the arterial wall, including smooth muscle cell proliferation and the initiation of atherosclerotic plaque development [5,6].

This form of vascular impairment is often systemic, affecting not only areas with manifest atherosclerosis but also peripheral vascular regions that appear clinically unaffected. It has been observed in individuals with a genetic predisposition to early cardiovascular disease (CVD), even in the absence of traditional risk factors. Other populations frequently exhibiting endothelial dysfunction include those with hyperlipidemia—specifically elevated low-density lipoprotein (LDL) and HDL levels—smokers, individuals with central obesity despite minimal coronary artery disease, and patients displaying insulin resistance or a family history of type 2 diabetes. Furthermore, it has been noted in cases of cardiac syndrome X and among older adults regardless of other health conditions. Psychological stress is also implicated, likely mediated through endothelin pathways. The extent and progression of endothelial dysfunction are closely tied to the severity and persistence of established risk factors, as well as the individual’s overall cardiovascular risk profile [5,6,7,8,9]. Understanding the mechanisms underlying endothelial dysfunction is therefore crucial for the early diagnosis, prevention, and treatment of CVDs. Addressing this topic not only provides deeper insight into the pathogenesis of CVDs but also highlights potential therapeutic targets.

## 2. The Endothelium and Endothelial NO Synthase: Structure, Function, and Physiological Significance

### 2.1. Structure and Functions of the Endothelium

The endothelium is considered a monocellular layer that separates all tissues from the circulating blood. Endothelial cells (ECs) are absent in invertebrates, cephalochordates, and tunicates but are present in the three major groups of extant vertebrates: hagfish, lampreys, and jawed vertebrates [10]. Blood vessel walls act as a selective barrier regulating molecule exchange between blood and tissues. The endothelial layer provides a large surface area—around 350 m^2^ in humans—for this exchange. It consists of a continuous monolayer of cells connected by adhesive structures called cell junctions, which include tight junctions, adherent junctions, and gap junctions. These junctions are made of transmembrane proteins linked to cytoskeletal networks. A physiological rise in blood flow enhances the hemodynamic forces exerted on the vessel wall, including shear stress—a tangential frictional force along the surface of ECs in the direction of blood flow—and pressure-induced stretch, which acts perpendicular to the vessel wall affecting both endothelial and smooth muscle cells. At rest, ECs typically have a polygonal shape, but as shear stress increases, they gradually align and elongate in the direction of the flow. This realignment helps to streamline the cells, reducing vascular resistance and potentially playing a key role in how blood vessels adapt to shear stress [11]. The endothelium functions not only as a physical barrier between the bloodstream and surrounding tissues, but also as an active regulator of vascular tone through the secretion of signaling molecules. ECs help maintain proper blood pressure and flow by producing both vasodilators—such as NO and prostacyclin (PGI_2_)—and vasoconstrictors—such as endothelin (ET) and platelet-activating factor (PAF). These mediators are not stored within cellular granules; instead, their biological effects depend largely on the distribution of specific receptors on vascular cells, their rapid breakdown, and gene-level control of their synthesis. While NO is continuously produced under normal conditions, its levels can be influenced by various external chemical and mechanical signals. In contrast, the production of prostaglandin I2, ET, and PAF are mainly triggered by changes in the extracellular environment [12].

### 2.2. Endothelial NO Synthase and Endothelium-Derived Hyperpolarizing Factor in Vascular Tone Regulation

Endothelial NO Synthase (eNOS) is encoded by the NOS3 gene in human beings. The eNOS enzyme protects the cardiovascular system (CVS), because of its ability to produce one of the most important neuromodulators in a proportionate amount that is physiologically beneficial to the CVS. NO is widely known as an autocrine and paracrine signaling factor that exerts numerous pleiotropic functions, such as modulation of blood flow and circulation, thrombosis, inflammation, immune regulation, as well as neural activity [13].

Within the endothelium, the amino acid L-arginine is transformed into L-citrulline and NO by one of the three NOS isoforms, specifically the endothelial isoform—eNOS. Quantitatively, shear stress and cell proliferation are considered the two primary regulators of eNOS gene expression. Nonetheless, eNOS is predominantly controlled through changes in its enzymatic activity. Activation of certain receptors by various agonists—such as bradykinin, serotonin, adenosine, ADP/ATP, histamine, and thrombin—enhances eNOS activity, at least partially by elevating intracellular free calcium (Ca^2+^) levels. Still, the mechanical force of shear stress remains the principal activator of eNOS, although the exact molecular pathways responsible for enzyme activation are yet to be fully understood [14].

NO produced by ECs with eNOS promotes vasodilation by diffusing into adjacent smooth muscle cells and activating guanylyl cyclase. NO diffuses into nearby vascular smooth muscle cells (SMCs), where it activates the enzyme soluble guanylyl cyclase. This activation triggers the production of cyclic guanosine monophosphate (cGMP), a second messenger that initiates a signaling cascade. One key outcome of this cascade is the activation of protein kinase G, which then phosphorylates various target proteins. These modifications lower intracellular calcium (Ca^2+^) levels in SMCs, promoting muscle relaxation and resulting in vasodilation. In addition to NO signaling, ECs can influence vascular tone by causing membrane hyperpolarization through the opening of potassium (K^+^) channels. Because ECs are electrically coupled to SMCs via myoendothelial gap junctions, this hyperpolarization spreads to the smooth muscle, causing the closure of voltage-dependent calcium channels. This further reduces Ca^2+^ entry into SMCs and reinforces vasodilation. In this way, ECs play a critical role in dynamically regulating vascular tone and blood vessel diameter through both chemical and electrical signaling mechanisms [15,16].

Current evidence suggests that NO generated by perivascular adipose tissue (PVAT) may facilitate vasorelaxation through several pathways: by diffusing into nearby smooth muscle cells and stimulating guanylyl cyclase, by promoting the release of adiponectin from PVAT adipocytes, or by affecting BKCa channels (large-conductance calcium-activated potassium channels) in smooth muscle cells, thereby enhancing membrane hyperpolarization [17]. The critical role of eNOS-derived NO in regulating vascular tone is supported by both animal and human studies, which have shown that eNOS inhibitors based on L-arginine lead to elevated blood or perfusion pressure, increased vascular resistance, and reduced blood flow in both in vivo and in vitro settings [18].

Another important mechanism of endothelial cell hyperpolarization is mediated by the factor Endothelium-Derived Hyperpolarizing Factor (EDHF), which plays a key role in vascular relaxation. This process begins with an increase in intracellular calcium concentration (Ca^2+^) in endothelial cells, triggered by activation of G protein–coupled receptors or agents such as calcium ionophores, thapsigargin, and cyclopiazonic acid. The rise in calcium activates calcium-sensitive potassium channels—SKCa (Small-Conductance Calcium-Activated Potassium Channels) and IKCa (Intermediate-conductance calcium-activated potassium)—located on the endothelial cell membrane. These channels initiate endothelial hyperpolarization, which is then transmitted to the vascular smooth muscle cells, causing their relaxation. Signal transmission occurs via two main pathways: directly through gap junctions or indirectly through the release of potassium ions into the intercellular space, where they activate potassium channels or the Na^+^/K^+^-ATPase in smooth muscle cells. Importantly, activation of SKCa and IKCa channels alone—even without a significant increase in calcium—can trigger an EDHF-mediated response. This indicates that endothelial hyperpolarization is the critical step underlying the relaxation of vascular smooth muscle [19].

## 3. Mechanisms of Endothelial Dysfunction

### 3.1. Endothelial Dysfunction vs. Endothelial Activation

All blood arteries’ inner surface is lined by endothelium, which is subject to changes in circulating metabolites that might impair vasodilator action. The generation of NO, which minimizes thrombosis, improves vascular compliance, encourages vasorelaxation, and regulates inflammation, is a vital role of the healthy endothelium [20]. The vascular endothelium is a dynamic and multifunctional monolayer lining all blood vessels, serving as a crucial regulator of vascular tone, permeability, hemostasis, and inflammation. Under physiological conditions, ECs maintain vascular homeostasis largely through the production of NO, a potent vasodilator synthesized by eNOS. NO not only promotes vasorelaxation and regulates blood flow but also possesses anti-thrombotic, anti-inflammatory, and anti-proliferative properties. However, endothelial function is highly sensitive to metabolic and inflammatory changes in the local environment [21]. Endothelial activation is a rapid and reversible response of ECs to acute inflammatory stimuli such as cytokines (e.g., TNF-α, IL-1β), chemokines (e.g., MCP-1), lipopolysaccharides, and oxidized LDL. In this state, ECs increase their expression of cell adhesion molecules (CAMs), including E-selectin, VCAM-1, and ICAM-1, which mediate leukocyte recruitment to sites of tissue injury or infection. Initially, selectins (especially E-selectin and P-selectin) promote the rolling of leukocytes along the endothelial surface [22]. This is followed by firm adhesion via interactions between leukocyte integrins (e.g., LFA-1, VLA-4) and endothelial immunoglobulin superfamily members such as ICAM-1 and VCAM-1. These interactions facilitate transendothelial migration of leukocytes into tissues where they exert immune functions [23]. Leukocyte adhesion to the vascular endothelium is a key step in their extravasation during inflammation, mediated primarily by E-selectin. Firm adhesion and transendothelial migration involve interactions between endothelial adhesion molecules—ICAM-1, VCAM-1—and leukocyte integrins such as LFA-1 (CD11a/CD18) and VLA-4 (CD49/CD29) [24] These adhesion molecules can also exist in soluble form in the circulation, reflecting endothelial activation. Once the stimulus is resolved, CAM expression diminishes, and ECs return to a resting state, preserving vascular integrity [25]. In contrast, endothelial dysfunction is a sustained and pathological state, often triggered by chronic exposure to cardiovascular risk factors such as oxidative stress, hyperglycemia, dyslipidemia, hypertension, and proinflammatory cytokines [26]. Unlike transient activation, dysfunction reflects a loss of protective endothelial functions, including impaired NO production due to eNOS uncoupling, increased oxidative stress, and peroxynitrite formation, which further reduces NO bioavailability and promotes vascular damage. This dysfunctional state is characterized by persistent upregulation of CAMs, especially ICAM-1 and VCAM-1, leading to chronic leukocyte adhesion and infiltration. These events contribute to vascular inflammation, remodeling, and progression of atherosclerotic lesions [27]. Moreover, in chronic inflammatory and metabolic diseases such as diabetes, obesity, and atherosclerosis, CAM expression remains elevated even in the absence of acute stimuli. Elevated plasma levels of soluble CAMs—particularly E-selectin, VCAM-1, and ICAM-1—are recognized biomarkers of endothelial dysfunction and have been correlated with disease severity and cardiovascular events [28].

### 3.2. Oxidative Stress and NO Pathway Impairment

**Figure 1:**
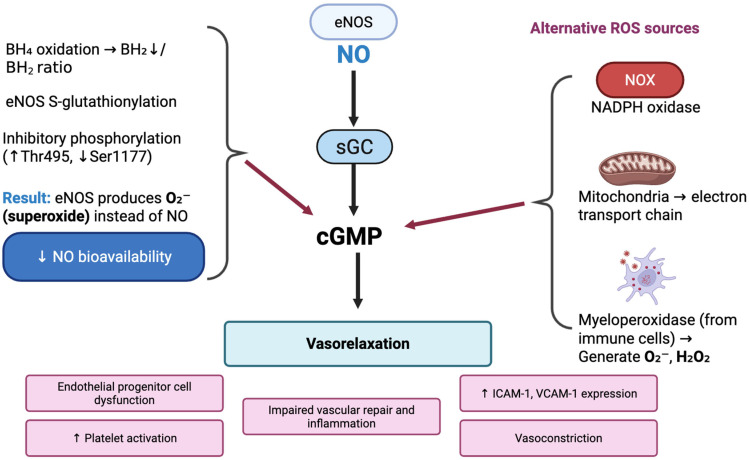
Impact of oxidative stress on nitric oxide (NO) signaling in vascular endothelium. BH_4_, tetrahydrobiopterin; cGMP, cyclic guanosine monophosphate; eNOS, endothelial nitric oxide synthase; H_2_O_2_, hydrogen peroxide; ICAM-1, intercellular adhesion molecule-1; MPO, myeloperoxidase; NO, nitric oxide; NOX, NADPH oxidase; O_2_^−^, superoxide anion; ROS, reactive oxygen species; sGC, soluble guanylate cyclase; VCAM-1, vascular cell adhesion molecule-1.

Oxidative stress, characterized by an imbalance between reactive oxygen species (ROS) production and antioxidant defenses, is a central contributor to endothelial dysfunction and impaired NO signaling in CVDs. Oxidative stress plays a central role in the pathogenesis of endothelial dysfunction and vascular diseases by disrupting the NO signaling pathway. Under normal physiological conditions, eNOS catalyzes the production of NO from L-arginine, a reaction that requires oxygen and the essential cofactor tetrahydrobiopterin (BH_4_) [29]. NO, in turn, exerts several protective vascular effects, including vasodilation, inhibition of leukocyte adhesion, suppression of smooth muscle cell proliferation, and prevention of platelet aggregation. However, this finely tuned system becomes highly susceptible to damage in the presence of oxidative stress, a condition characterized by an excess of ROS such as superoxide anion (O_2_^−^), hydrogen peroxide (H_2_O_2_), and hydroxyl radicals [30]. A key consequence of oxidative stress is the oxidation of BH_4_ to its inactive oxidized form, dihydrobiopterin (BH_2_), which destabilizes the eNOS dimer and leads to a phenomenon known as “eNOS uncoupling.” In this uncoupled state, eNOS loses its ability to produce NO and instead becomes a source of superoxide itself (Figure 1). This shift in enzymatic function not only diminishes NO bioavailability but also increases oxidative stress within the vascular wall, creating a self-reinforcing cycle of ROS generation and endothelial injury. The superoxide produced in this process can rapidly react with any remaining NO to form peroxynitrite (ONOO^−^), a potent oxidant that exacerbates endothelial damage by oxidizing lipids, proteins, and DNA. Peroxynitrite further oxidizes BH_4_, thereby perpetuating eNOS uncoupling and leading to sustained NO deficiency. In addition to depleting cofactors, oxidative stress interferes with the regulatory phosphorylation of eNOS. Under healthy conditions, phosphorylation at Ser^1177^ by kinases such as Akt enhances eNOS activity, while phosphorylation at Thr^495^ reduces NO synthesis by preventing eNOS-calmodulin interaction. ROS and oxLDL inhibit the PI3K/Akt signaling pathway, leading to reduced Ser^1177^ phosphorylation and a corresponding increase in Thr^495^ phosphorylation. This shift contributes to a functional inactivation of eNOS, compounding the impact of cofactor loss. Moreover, inflammatory cytokines and ROS promote the activation of protein kinase C (PKC), which can further phosphorylate eNOS at inhibitory sites, intensifying the reduction in NO production [31]. Another significant mechanism by which oxidative stress disrupts NO signaling involves post-translational modifications of eNOS, particularly S-glutathionylation. Under conditions of redox imbalance, oxidized glutathione reacts with specific cysteine residues on eNOS, modifying its structure and favoring a state that promotes superoxide rather than NO production, even when eNOS remains dimerized. This form of redox-induced functional uncoupling highlights how ROS can impair eNOS activity independently of dimer disruption or BH_4_ oxidation. Such modifications are reversible, suggesting potential therapeutic targets, yet in chronic oxidative conditions, sustained S-glutathionylation may lead to long-term eNOS dysfunction [29].

The reduction in NO bioavailability has widespread implications for vascular health. NO is a critical regulator of vascular tone; its absence leads to increased vasoconstriction, elevated blood pressure, and reduced tissue perfusion. Furthermore, NO normally inhibits the expression of endothelial adhesion molecules such as ICAM-1 and VCAM-1 and suppresses leukocyte adhesion and transmigration. When NO levels decline, ECs become more adhesive to leukocytes, enhancing vascular inflammation and promoting the initiation and progression of atherosclerotic lesions. Platelet activation is also heightened in NO-deficient states, contributing to thrombosis and increasing the risk of acute cardiovascular events such as myocardial infarction or stroke [30].

Oxidative stress–induced NO disruption also affects the downstream signaling of NO through its receptor, soluble guanylate cyclase (sGC). NO activates sGC, which catalyzes the conversion of GTP to cGMP, leading to smooth muscle relaxation and vasodilation. However, peroxynitrite and other ROS can oxidize the heme group in sGC, rendering it less responsive or unresponsive to NO stimulation. This impairs cGMP production and disrupts the normal vasodilatory response, even in the presence of NO, indicating that oxidative stress can also produce resistance at the level of signal transduction [29].

Beyond its vascular effects, oxidative stress and NO pathway impairment play critical roles in metabolic diseases such as diabetes, where hyperglycemia increases mitochondrial ROS generation and activates NADPH oxidase. In such conditions, endothelial dysfunction becomes a key mediator of microvascular and macrovascular complications. Similarly, in hypertension, angiotensin II stimulates ROS production through NADPH oxidase, promoting eNOS uncoupling and vascular remodeling. Ischemia-reperfusion injury is another context in which a surge of ROS during reperfusion severely impairs eNOS function, compounding tissue damage [32].

Given these mechanisms, targeting oxidative stress and eNOS dysfunction offers promising therapeutic avenues. Antioxidants such as vitamin C, vitamin E, and polyphenols have been investigated for their capacity to scavenge ROS and preserve NO bioavailability, with mixed clinical results. More targeted interventions, including BH_4_ supplementation or agents that stabilize eNOS dimers, have shown efficacy in experimental models but remain under evaluation in clinical settings [33]. Pharmacologic activation of sGC with NO-independent stimulators or stabilizers (e.g., riociguat) is another approach to restore cGMP signaling in states of NO resistance. Moreover, interventions that upregulate endogenous antioxidant defenses, such as Nrf2 activators, may offer a broader protective effect by enhancing cellular resilience to oxidative damage [34]. In conclusion, oxidative stress impairs the NO pathway through a complex interplay of molecular disruptions, including BH_4_ oxidation and eNOS uncoupling, peroxynitrite formation, adverse phosphorylation and post-translational modifications of eNOS, and downstream resistance at the level of sGC. These alterations collectively diminish NO bioavailability and activity, leading to endothelial dysfunction, vascular inflammation, and heightened cardiovascular risk. Understanding these mechanisms not only elucidates the pathophysiology of vascular disease but also identifies potential therapeutic targets to restore NO signaling and improve vascular health in oxidative stress-related conditions [35].

Flavonoids (dietary polyphenols found in fruits, vegetables, tea and cocoa) exert multiple actions that may protect the endothelium: direct ROS scavenging, inhibition of NADPH oxidase, preservation of BH_4_ and eNOS coupling, upregulation of endothelial antioxidant defenses (e.g., Nrf2 pathway), and enhancement of eNOS phosphorylation/activation. In humans, flavonoid-rich diets or supplements have shown improvements in surrogate measures such as flow-mediated dilation in several trials, but heterogeneity in compound classes, bioavailability, dosing, and endpoints complicates interpretation. Thus, flavonoids are mechanistically attractive, yet rigorous randomized trials with well-defined preparations and clinical endpoints are still needed to translate experimental benefits into recommendations [36,37].

The translational gap between experimental models and clinical trials in endothelial-targeted therapies reflects multiple factors. Preclinical models (cell culture, small rodents) often have simplified pathophysiology, shorter disease timelines, and different drug metabolism compared with human chronic cardiometabolic disease [38]. Many antioxidant or eNOS-targeting interventions show clear mechanistic benefit in vitro or in animal models but fail in the clinic because of inadequate bioavailability, off-target effects, inappropriate dosing/timing (too late in disease course), or reliance on surrogate endpoints that do not predict hard clinical outcomes. Moreover, patient heterogeneity (comorbidities, concomitant drugs, genetic background) and the complexity of human inflammatory networks blunt single-target strategies [39].

### 3.3. Inflammatory Pathways and Mediators

Endothelial dysfunction is intricately linked to chronic low-grade inflammation, with cytokines and chemokines playing pivotal roles in mediating vascular injury and immune cell recruitment. Among the most prominent inflammatory mediators are tumor necrosis factor-alpha (TNF-α), interleukin-6 (IL-6), and monocyte chemoattractant protein-1 (MCP-1), which act both locally and systemically to promote endothelial activation and injury [40]. TNF-α, a key proinflammatory cytokine produced by activated macrophages, ECs, and adipocytes, initiates and amplifies vascular inflammation [41]. It induces the expression of adhesion molecules such as ICAM-1 and VCAM-1 via activation of nuclear factor-κB (NF-κB), thereby enhancing leukocyte adhesion and transmigration into the vascular wall. Furthermore, TNF-α stimulates ROS generation and disrupts eNOS activity, contributing to NO depletion and promoting oxidative stress. IL-6, another pleiotropic cytokine elevated in various cardiometabolic disorders, exacerbates endothelial dysfunction by increasing vascular permeability, promoting leukocyte recruitment, and perpetuating inflammatory signaling cascades. Chronic IL-6 exposure impairs endothelial regeneration and fosters a pro-thrombotic state through upregulation of fibrinogen and plasminogen activator inhibitor-1 (PAI-1) [42]. MCP-1, a chemokine secreted in response to oxidative and inflammatory stimuli, is instrumental in monocyte recruitment to sites of endothelial injury. Binding to its receptor CCR2 on monocytes, MCP-1 promotes their transmigration and differentiation into macrophages, which further propagate inflammation through cytokine production and ROS release [43]. Elevated levels of these mediators have been correlated with impaired flow-mediated dilation (FMD), increased arterial stiffness, and progression of atherosclerotic lesions [44]. Collectively, these inflammatory pathways form a self-reinforcing loop, wherein cytokine- and chemokine-induced endothelial dysfunction amplifies immune activation, fostering vascular inflammation, remodeling, and ultimately, cardiometabolic disease [45].

Chronic and persistent inflammation, together with sustained oxidative stress not only injure mature endothelial cells (ECs) but also markedly impair endogenous vascular regenerative mechanisms. Endothelial progenitor cells (EPCs) and endothelial colony-forming cells (ECFCs) show reduced mobilization, proliferative capacity, and increased senescence in the context of chronic inflammatory states and redox imbalance [46,47]. Mechanisms include proinflammatory cytokine–driven NF-κB activation, mitochondrial dysfunction, telomere shortening, and oxidative damage to DNA and proteins within progenitor pools, which together blunt homing signals (e.g., SDF-1/CXCR4), reduce nitric oxide–mediated mobilization, and impair angiogenic signaling. As a result, re-endothelialization after vascular injury is delayed, repair is incomplete, and maladaptive remodeling (intimal hyperplasia, fibrosis) is promoted—processes that link endothelial dysfunction to progressive vascular disease [48]. Therapeutic strategies that target inflammation, restore redox balance (for example, by supporting BH_4_ levels or enhancing Nrf2-dependent antioxidant responses) and rescue EPC function are therefore an active area of translational research [49].

### 3.4. The Impact of Adipocytokines and Chemokines

Adipose tissue is increasingly recognized as an active endocrine organ that secretes a variety of bioactive molecules—collectively known as adipokines—which modulate endothelial function and systemic inflammation. Among these, leptin, adiponectin, and resistin have garnered significant attention for their divergent roles in vascular homeostasis [50]. Leptin, a hormone primarily involved in appetite regulation, exhibits proinflammatory and pro-atherogenic properties in the vascular context [51]. Elevated in obesity and metabolic syndrome, leptin promotes endothelial dysfunction through increased oxidative stress, eNOS inhibition, and upregulation of adhesion molecules. It also enhances the production of proinflammatory cytokines such as IL-6 and TNF-α, further contributing to vascular inflammation [52]. Additionally, leptin activates the Janus kinase/signal transducers and activators of transcription (JAK/STAT) pathway in ECs, promoting cellular proliferation and migration, which may contribute to vascular remodeling and neointima formation [51]. Conversely, adiponectin is a vasculoprotective adipokine that exerts anti-inflammatory, anti-oxidative, and insulin-sensitizing effects. It enhances eNOS activity and NO bioavailability, inhibits NF-κB signaling, and suppresses the expression of adhesion molecules and inflammatory cytokines [52,53,54]. Hypoadiponectinemia—commonly observed in obesity, insulin resistance, and type 2 diabetes—is associated with impaired endothelial function and heightened cardiovascular risk [55,56]. Resistin, another adipokine predominantly produced by macrophages in humans, acts as a proinflammatory mediator by promoting the expression of ICAM-1, VCAM-1, and MCP-1 via NF-κB activation [57]. It also impairs insulin signaling and induces oxidative stress, thereby contributing to both metabolic dysregulation and vascular injury [58]. Elevated resistin levels correlate with endothelial dysfunction and are predictive of atherosclerotic progression [59]. Taken together, the balance between protective adipokines like adiponectin and deleterious ones like leptin and resistin plays a critical role in the regulation of endothelial function. Disruption of this balance in obesity and related metabolic disorders leads to a proinflammatory, pro-oxidative vascular milieu, facilitating the development of cardiometabolic complications [60].

Emerging evidence links gut microbiota composition (dysbiosis) to endothelial function via several mechanistic routes. Microbial metabolites such as trimethylamine-N-oxide (TMAO) increase vascular inflammation and activate inflammasomes (e.g., NLRP3), promote oxidative stress and endothelial adhesion-molecule expression, and have been associated epidemiologically with impaired flow-mediated dilation and atherosclerotic risk [61,62]. Bacterial products (e.g., lipopolysaccharide) translocating across a leaky gut barrier can drive systemic low-grade inflammation, activating endothelial NF-κB signaling and NADPH oxidases, thereby reducing NO bioavailability [63]. Although causal pathways are still being defined, modulation of the gut–heart axis (dietary interventions, microbiome-targeted therapies) represents a promising avenue to modify endothelial health. Given rapid progress in the field, it is advisable to cite both mechanistic studies and recent reviews when discussing microbiome–endothelium interactions [64].

## 4. Endothelial Dysfunction in Cardiometabolic Diseases

### 4.1. Diabetes and Insulin Resistance

Diabetes mellitus (DM) is a global health challenge that significantly contributes to the burden of CVD. It is strongly linked with an elevated risk of both microvascular complications—such as retinopathy, nephropathy, and neuropathy—and macrovascular conditions, including atherosclerosis. Notably, individuals with DM have a two- to fourfold higher risk of developing CVD compared to those without the condition, highlighting the profound systemic impact of dysregulated glucose metabolism [65].

**Figure 2:**
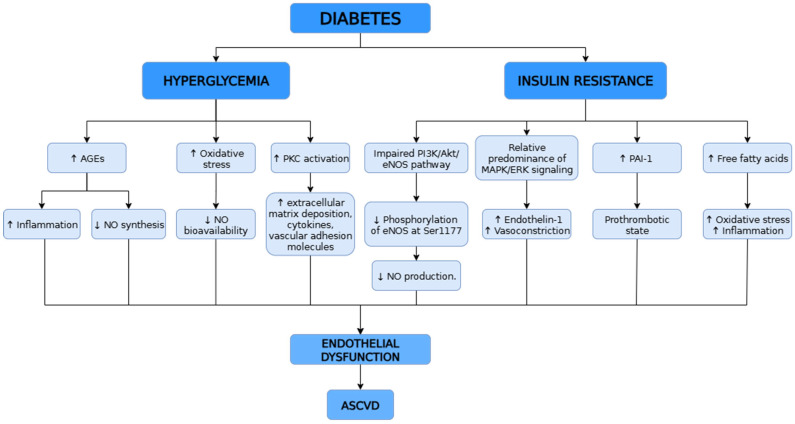
Mechanisms of endothelial dysfunction in type 2 diabetes mellitus (T2DM). AGEs—advanced glycation end products, PKC—protein kinase C, PI3K/Akt/eNOS—phosphoinositide 3-Kinase/endothelial nitric oxide synthase, PAI-1—plasminogen activator inhibitor-1, NO—nitric oxide, ↑—increase, ↓—decrease.

Macrovascular complications in the type II DM (T2DM) affect large vessels and include atherosclerotic cardiovascular diseases (ASCVD) such as coronary artery disease, cerebrovascular disease, and peripheral artery disease. A key mechanism underlying these complications is endothelial dysfunction, which plays a central role in the development and progression of ASCVD (Figure 2). Both animal and human studies have demonstrated that endothelial dysfunction is significantly present in DM and correlates with adverse clinical outcomes, highlighting its role as a major contributor to diabetic vascular disease [66]. The endothelium regulates vascular tone through the release of vasodilators such as NO and PGI_2_, as well as vasoconstrictors like endothelin-1 (ET-1) and angiotensin II. In DM, chronic hyperglycemia impairs endothelial function primarily by reducing NO bioavailability and increasing oxidative stress. Additionally, hyperglycemia promotes the formation of advanced glycation end products (AGEs), which exacerbate inflammation and further suppress NO synthesis. Activation of PKC leads to increased production of extracellular matrix components, cytokines, and vascular adhesion molecules, contributing to inflammation, enhanced permeability, and abnormal vascular cell proliferation. Hyperglycemia also activates nuclear inflammation by production of vascular adhesion molecules and cytokines [67,68].

Endothelial injury does not only occur because of high levels of glucose, but also because of high levels of insulin due to insulin resistance [67]. Under physiological conditions, insulin stimulates the phosphatidylinositol 3-kinase (PI3K) signaling pathway, which leads to the phosphorylation of endothelial eNOS at Ser1177 in ECs, promoting NO production and maintaining vascular homeostasis. However, in insulin resistance, this signaling cascade is impaired, resulting in a dysregulation between the PI3K/Akt and MAPK/ERK pathways. This imbalance contributes to reduced NO synthesis and endothelial dysfunction [69]. Insulin resistance increases expression of PAI-1 that has a part in the coagulation system. Excessive release of free fatty acids due to insulin resistance leads to increased vascular inflammation and expression of NADPH oxidases, mitochondrial uncoupling and increased superoxide production [67].

DM, especially T2DM, significantly increases cardiovascular risk, largely through endothelial dysfunction. Chronic hyperglycemia reduces NO availability, increases oxidative stress, and promotes inflammation via AGEs and protein kinase C activation. Insulin resistance further impairs endothelial function by disrupting PI3K/Akt/eNOS signaling, enhancing proinflammatory pathways, and increasing oxidative stress through elevated free fatty acids. These mechanisms together drive the development and progression of ASCVD in DM.

### 4.2. Dyslipidemia and Atherosclerosis

**Figure 3:**
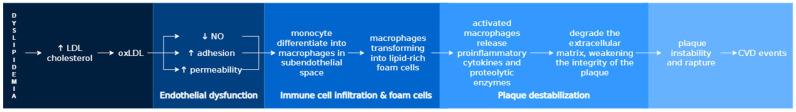
Mechanisms of endothelial dysfunction and atherogenesis in dyslipidemia. LDL—low-density lipoprotein, oxLDL—oxidized LDL, NO—nitric oxide, CVD—cardiovascular disease, ↑—increase, ↓—decrease.

Endothelial dysfunction is recognized as an early and essential factor in the initiation and progression of atherosclerosis, ultimately contributing to cardiovascular complications. Dyslipidemia, particularly elevated LDL cholesterol, further worsens endothelial function when poorly controlled, and over time, this dysfunction becomes a key driver in the development of serious cardiovascular events such as myocardial infarction and stroke [70]. Oxidized LDL (oxLDL) is considered a key factor in initiating endothelial dysfunction and vascular inflammation that promote atherosclerosis (Figure 3), although the exact mechanisms are not yet fully understood. Research shows that oxLDL triggers several harmful effects, including the release of proinflammatory cytokines, increased expression of adhesion molecules, stimulation of monocyte chemotactic protein-1, and promotion of smooth muscle cell proliferation. Moreover, oxLDL impairs endothelium-dependent vasodilation and disrupts NO synthase (NOS) activity. This imbalance reduces eNOS function and enhances inducible NOS expression, further amplifying vascular inflammation and accelerating atherosclerotic changes [71]. When the endothelium becomes dysfunctional, its ability to induce vasodilation is reduced, leading to impaired barrier function, increased vascular permeability, and elevated expression of leukocyte adhesion molecules. This promotes monocyte adhesion and migration into the subendothelial space, where they differentiate into macrophages. These macrophages internalize oxLDL through scavenger receptors such as CD36, lectin-like oxidized LDL receptor-1 (LOX-1), and SR-A1, transforming into lipid-rich foam cells [72]. These receptors play a key role in identifying and clearing abnormal intracellular substances, such as oxidized LDL generated by oxidative stress and glycated proteins. The main receptors in this group include scavenger receptor class A (SR-A) and LOX-1. SR-A facilitates the uptake of oxidized LDL into ECs, while LOX-1 recognizes both oxidized LDL and glycated proteins. These receptors are closely linked to the development of atherosclerosis and CVD, suggesting that targeting their activity may offer a promising therapeutic strategy [70]. OxLDL is also taken up by vascular smooth muscle cells, which can similarly become foam cells. Additionally, oxLDL influences VSMC behavior by promoting their migration, proliferation, calcification, phenotypic switching from contractile to synthetic type, and apoptosis [72]. As a result of macrophage activation within the vessel wall, a range of proinflammatory cytokines is released, along with the generation of ROS and proteolytic enzymes. These enzymes degrade the extracellular matrix, weakening the structural integrity of the atherosclerotic plaque. This process contributes to plaque instability, increasing the risk of rupture and subsequent thrombotic cardiovascular events. Additionally, the colocalization of LOX-1 and the pro-apoptotic 2protein Bax in human atherosclerotic plaques—especially in the vulnerable shoulder regions prone to rupture—further supports the role of LOX-1 in promoting plaque destabilization. This suggests that LOX-1 may contribute not only to foam cell formation but also to processes that weaken plaque stability and increase the risk of acute cardiovascular events [73]. Growing evidence highlights the crucial role of monocytes and macrophages—key cells of the innate immune system—in the early stages of atherogenesis. Monocyte recruitment to the arterial wall begins early and continues even in advanced atherosclerotic lesions. This process involves adhesion to activated ECs and migration into the intima, guided by chemokines. Once inside, monocytes mature into macrophages, proliferate, and release proinflammatory mediators.

Recent findings show that hyperlipidemia increases a proinflammatory monocyte subset (Ly-6C^high in mice, possibly PSGL^+ in humans) that actively contributes to lesion progression by producing cytokines and matrix-degrading enzymes. Additionally, mast cells may also play a role in atherosclerosis by releasing vasoactive substances and proteases that promote inflammation and vascular remodeling [74].

Endothelial dysfunction is a key early event in atherogenesis, worsened by dyslipidemia and oxLDL, which promotes inflammation, impairs NO signaling, and disrupts vascular homeostasis. OxLDL is taken up by macrophages and smooth muscle cells via scavenger receptors like LOX-1 and SR-A, leading to foam cell formation and plaque development. Activated macrophages release inflammatory mediators and enzymes that weaken the plaque structure, increasing the risk of rupture. Hyperlipidemia also drives proinflammatory monocyte recruitment, while mast cells contribute to vascular inflammation and remodeling, further advancing atherosclerosis.

### 4.3. Hypertension and Obesity

**Figure 4:**
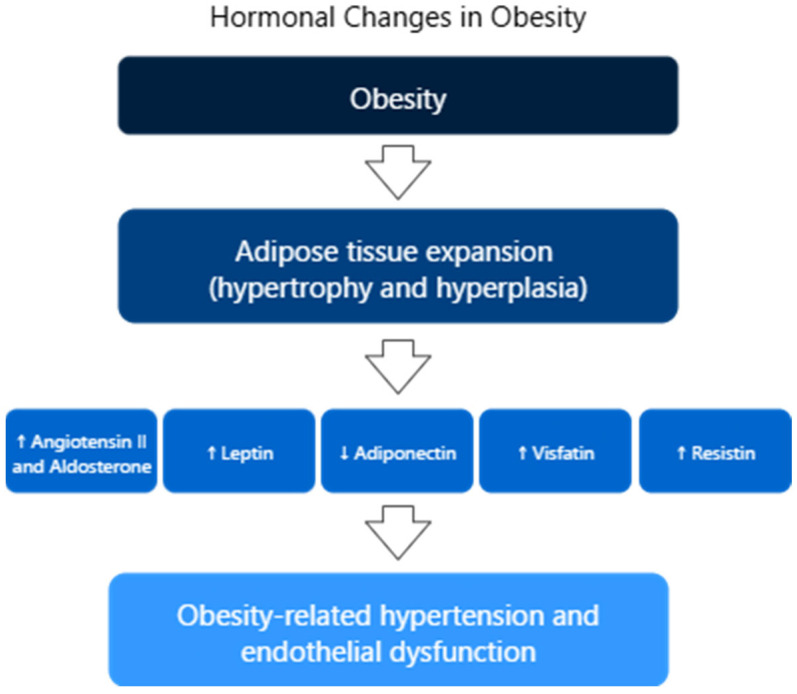
Hormonal changes in obesity leading to hypertension and endothelial dysfunction. ↑—increase, ↓—decrease.

Hypertension remains a leading cause of cardiovascular complications and increased mortality. Recent research highlights the crucial role of endothelial dysfunction in the onset of arterial hypertension. Endothelial dysfunction is considered a characteristic feature of the vascular system in individuals with hypertension. Additionally, studies have shown that endothelial dysfunction contributes to the development of both atherosclerosis and arteriosclerosis. A key factor in this process is the reduced bioavailability of NO, which is essential for proper vascular function. Other contributing elements include oxidative stress and vascular inflammation, which influence vascular resistance through two main mechanisms: (1) impaired vasoconstriction and vasodilation, and (2) stimulation of vascular smooth muscle cell proliferation and the initiation of arteriosclerotic processes. The activation of pro-by-inflammatory, pro-fibrotic, redox-sensitive, and growth/apoptosis-related pathways leads to structural, functional, and mechanical alterations in blood vessels, resulting in remodeling, calcification, and further endothelial dysfunction [75]. In hypertension, the persistent elevation of pressure in small blood vessels causes premature aging and increased turnover of ECs. These damaged cells are replaced by newly regenerated ones, but the new endothelium has a reduced ability to produce endothelium-derived relaxing factors (EDRFs), especially NO. As a result, there is a shift in balance toward endothelium-derived contracting factors (EDCFs), such as angiotensin II, endothelins, uridine adenosine tetraphosphate, and cyclooxygenase-derived substances, leading to increased vasoconstriction [76,77]. For example, endothelin-1 is the main isoform produced by ECs, is a strong vasoconstrictor that raises blood pressure by promoting smooth muscle contraction and sodium retention. It acts through ETA and ETB receptors on vascular smooth muscle, triggering calcium release via IP3. ETB receptors on ECs, however, stimulate NO release and promote natriuresis, temporarily lowering blood pressure. Systemic administration of endothelin-1 first causes brief vasodilation (via endothelial ETB), followed by prolonged vasoconstriction (via ETA and ETB on smooth muscle). Endothelin-1 also promotes the release of cyclooxygenase-derived EDCFs, like thromboxane A2, further enhancing vasoconstriction. This contributes to the imbalance in hypertension, where vasoconstrictors dominate due to impaired endothelial function [77]. Obesity contributes significantly to endothelial dysfunction and the development of hypertension by disrupting the normal function of PVAT. In healthy individuals, PVAT supports blood vessel relaxation by releasing vasodilatory adipokines. However, in obesity, this balance is disturbed—the secretion of relaxing factors decreases, while proinflammatory and vasoconstrictive substances like TNF-α and ET-1 increase. This shift leads to a reduced availability of NO and an imbalance between NO and ET-1 signaling, favoring vasoconstriction. Additionally, obesity is associated with increased oxidative stress, mainly due to activation of NADPH oxidase (NOX2), which leads to eNOS uncoupling. This process lowers NO production and increases superoxide generation, further impairing vascular relaxation. Interestingly, although overall ROS production may be lower in obese animals, their blood vessels are more sensitive to ROS, which intensifies the effects of ET-1. These mechanisms—combined with those described earlier, such as impaired NO release and increased EDCFs—contribute to the progression of endothelial dysfunction and obesity-related hypertension [78]. In obesity, adipose tissue releases hormones and factors that disrupt vascular function and raise blood pressure (Figure 4). Adipocytes produce angiotensin II and aldosterone, promoting vasoconstriction via vascular smooth muscle activation. Obesity leads to adipose tissue expansion through hypertrophy and hyperplasia, accompanied by increased levels of leptin. Elevated leptin stimulates sympathetic nervous system activity, raising heart rate and blood pressure. Blocking leptin or its receptors in the hypothalamus normalizes blood pressure in obese rodents. Interestingly, leptin-deficient animals and humans have lower blood pressure despite severe obesity, though the link between chronic high leptin levels, leptin resistance, and hypertension remains unclear. Adiponectin, another adipokine, is typically reduced in obesity—especially in individuals with visceral fat accumulation—and lower levels are associated with hypertension. Weight loss and antihypertensive treatments can increase adiponectin levels and improve blood pressure. Other adipokines like visfatin and resistin are also increased. Visfatin rises with hypoxia and is seen in hypertension, while resistin is associated with insulin resistance and hypertension in diabetics. These changes contribute to the development of obesity-related hypertension [79]. Hypertension is closely linked to endothelial dysfunction, marked by reduced NO and increased vasoconstrictors like angiotensin II and endothelin-1. This imbalance leads to vessel constriction, inflammation, and vascular remodeling. Obesity worsens this process by disrupting perivascular fat function, increasing inflammation and oxidative stress, and lowering NO availability. Elevated leptin and reduced adiponectin further raise blood pressure, contributing to obesity-related hypertension.

### 4.4. Chronic Low-Grade Inflammation as a Link Between Endothelial Dysfunction and Metabolic Disorders

As mentioned in the previous section, obesity is a chronic metabolic condition marked by low-grade inflammation and plays a key role in the development of endothelial dysfunction. In obesity, various factors—such as elevated LDL and triglyceride levels, oxidative stress, chronic inflammation, and hemodynamic imbalance—disrupt normal endothelial function. This leads to impaired NO and PGI_2_ production, reduced EDHF synthesis, and increased levels of vasoconstrictors like angiotensin II and prostaglandin H_2_. As a result, processes like leukocyte adhesion, platelet activation, atherosclerosis, and thrombosis are triggered. A central role is played by insulin resistance, oxLDL, inflammation in adipose tissue, and reduced NO bioavailability. In particular, visceral adipose tissue contributes significantly to inflammation and oxidative stress, further destabilizing the endothelium. Excess nutrients in adipose tissue stimulate the release of inflammatory mediators (e.g., IL-6, IL-1β, TNF-α, leptin) and MCP-1 while lowering adiponectin, creating a proinflammatory environment that promotes vascular dysfunction and atherosclerosis [80,81]. In addition, the role of inducible nitric oxide synthase (iNOS, NOS2) is crucial in inflammatory conditions linked to endothelial dysfunction. Chronic activation of iNOS leads to excessive NO production, which in the presence of superoxide generates peroxynitrite (ONOO^−^). This highly reactive oxidant amplifies oxidative and nitrosative stress, upregulates proinflammatory signaling pathways such as NF-κB, and increases the expression of adhesion molecules (ICAM-1, VCAM, P-selectin), thereby promoting leukocyte adhesion and vascular inflammation. ONOO^−^ also induces mitochondrial dysfunction, DNA and protein damage, and apoptosis, further reducing NO bioavailability and impairing vasodilation. Through these mechanisms, sustained iNOS activity contributes to vascular injury, chronic inflammation, and the progression of cardiometabolic complications including diabetes, hypertension, and atherosclerosis [82].

Arachidonic acid metabolites like thromboxane A2 and prostacyclin affect vascular tone—when imbalanced, they promote vasoconstriction and damage. Inflammatory signaling (e.g., PI3K/AKT, NF-κB) increases COX-2 and ROS production via NOX2, creating a cycle of oxidative stress and endothelial injury. Immune cells such as M1 macrophages, neutrophils, and dendritic cells release proinflammatory cytokines and ROS, form NETs, and activate inflammasomes like NLRP3, all contributing to chronic vascular inflammation. These immune responses play a key role in endothelial damage and vascular complications in metabolic diseases [83]. Interestingly ECs, which line the blood vessels, share a developmental origin with immune cells, suggesting they may also play roles in immune function. In fact, certain ECs—such as liver sinusoidal ECs—can originate from bone marrow precursors during liver damage or regeneration, similar to immune cells. Some EC subtypes go beyond their traditional vascular roles and actively participate in immune regulation, including immune tolerance, inflammation, and immune cell recruitment. These ECs can exhibit features typical of immune cells: they express immune receptors, secrete cytokines, induce apoptosis in other cells (e.g., through FASL), and act as antigen-presenting cells. Some can even perform phagocytosis and help clear dead cells (efferocytosis). Their immune activity is influenced by cytokines like IL-35 and IL-17A. Since ECs are among the first cells to encounter pathogens and interact with immune cells entering tissues, they are uniquely positioned to serve as an early defense mechanism in immune responses [84]. Endothelial function is further impaired by immune system activity. Inflammatory pathways, oxidative stress (via NOX2), and immune cells such as macrophages and neutrophils contribute to chronic vascular inflammation and damage. Additionally, ECs themselves can act like immune cells—presenting antigens, producing cytokines, and participating in immune responses—making them key players in vascular and immune system interactions, particularly in metabolic diseases like obesity and DM.

## 5. Biomarkers of Endothelial Dysfunction

Biomarkers, also referred to as biological markers, are defined as measurable indicators that reflect physiological, pathological, or pharmacological processes within the body [85,86]. As outlined by the National Institutes of Health, a biomarker is “a characteristic that is objectively measured and evaluated as an indicator of physiological processes, pathogenic processes, or pharmacologic responses to a therapeutic intervention” [87]. Similarly, the World Health Organization expands this definition to include any biological response that may indicate interaction between a biological system and a potentially hazardous agent of chemical, physical, or biological origin. Biomarkers encompass a wide spectrum of clinical parameters, ranging from vital signs such as blood pressure and heart rate to complex biochemical analyses of body fluids and tissues, and are increasingly recognized as essential tools in diagnosis, prognosis, and therapeutic monitoring [86]. In the context of vascular health, biomarkers specific to endothelial dysfunction provide critical insights into early vascular injury and the progression of cardiovascular and inflammatory diseases. Identifying biomarkers of inflammation and oxidative stress can aid clinicians in evaluating treatment effectiveness and support the development of novel therapies for individuals at risk of CVD. However, the multifactorial nature of CVD makes it unlikely that a single biomarker could reliably predict the absolute risk of future cardiovascular events [88].

### 5.1. Molecular and Biochemical Indicators

#### 5.1.1. Cell Adhesion Molecules

CAMs are essential regulators of cellular communication, tissue integrity, and homeostasis. They mediate cell-to-cell and cell-to-matrix interactions, while also participating in intracellular signaling through their connection with the cytoskeleton and adaptor proteins. CAMs contribute to the formation of endothelial and epithelial barriers by facilitating signal transduction and homotypic interactions at cellular junctions [89,90]. Upon activation, ECs increase the surface expression of various CAMs, including E-selectin, P-selectin, intercellular adhesion molecule-1 (ICAM-1), and vascular cell adhesion molecule-1 (VCAM-1) [91]. E-selectin and P-selectin facilitate the initial capture and rolling of monocytes along the endothelium, whereas ICAM-1 and VCAM-1 mediate stronger adhesive interactions that promote monocyte arrest and subsequent transendothelial migration. The functional significance of CAMs becomes most evident during the process of endothelial activation, which involves substantial alterations in endothelial cell behavior in response to inflammatory stimuli. This response is initiated by resident immune cells, such as tissue macrophages, which act as early sentinels by secreting proinflammatory cytokines. These mediators activate ECs and promote the release of chemokines that attract circulating leukocytes to the site of inflammation. In turn, activated ECs amplify the inflammatory response by producing additional cytokines and chemokines, thereby enhancing leukocyte recruitment and tissue infiltration. Under homeostatic conditions, leukocytes do not interact strongly with the endothelium; however, upon activation, ECs upregulate the expression of CAMs, enabling firm adhesion and subsequent transmigration of immune cells across the vascular wall [92].

ICAMs are surface glycoproteins expressed on numerous cell types and exhibit distinct regulatory and functional characteristics [93]. Among CAMs, ICAM-1 has received the most attention due to its broad expression profile and main involvement in inflammatory responses [94]. This transmembrane protein, encoded by the *ICAM1* gene located on chromosome 19, was first characterized in the 1980s as the primary ligand for the β2 integrin lymphocyte function-associated antigen-1 (LFA-1; CD11a/CD18), marking a key advancement in the understanding of leukocyte-endothelium interactions [94]. ICAM-1 is now recognized as a central mediator of leukocyte adhesion and transmigration during immune activation. Its expression is strongly inducible in ECs under proinflammatory conditions and is regulated by a range of cytokines and signaling molecules, including TNF-α, IL-1β, IL-6, IFN-γ, NF-κB, as well as oxidative stress mediators such as hydrogen peroxide and NADPH oxidase activity [94] (Table 1).

VCAM-1 is another adhesion molecule, primarily expressed on ECs and plays a key role in inflammation by mediating leukocyte adhesion and transendothelial migration. First identified in 1989, VCAM-1 contributes to the recruitment of immune cells such as T cells and macrophages at sites of vascular injury. While predominantly associated with ECs, its expression has also been observed in other cell types including dendritic cells, stromal cells, astrocytes, and macrophages.

VCAM-1 expression is upregulated in response to proinflammatory stimuli such as TNF-α, ROS, oxLDL, and shear stress. Upon activation, VCAM-1 binds to integrins on leukocytes, triggering intracellular signaling, cytoskeletal remodeling, and disruption of endothelial junctions, thereby facilitating leukocyte extravasation [95] (Table 1).

Soluble cell adhesion molecules (sCAMs) are circulating forms of membrane-bound CAMs that participate in key physiological and pathological processes. While direct assessment of CAMs expression on cells remains clinically challenging, sCAMs can be readily measured in serum or plasma, serving as potential surrogate markers of endothelial activation [96] (Table 1).

#### 5.1.2. Acute Phase Proteins and Inflammatory Cytokines

C-reactive protein (CRP) is a key acute-phase protein predominantly produced by hepatocytes in response to proinflammatory cytokines, including IL-1, IL-6, and TNF-α. Functionally, CRP contributes to endothelial dysfunction by suppressing eNOS gene expression, thereby reducing NO bioavailability. Numerous clinical investigations have demonstrated a strong association between elevated CRP concentrations and various stages of CAD, indicating its involvement in endothelial impairment [1]. High-sensitivity CRP levels, in particular, have been identified as independent markers of impaired coronary vasoreactivity in patients without obstructive coronary lesions, and have shown positive correlations with IL-6, LDL-cholesterol, and increased cardiovascular risk and mortality. Moreover, circulating levels of IL-6 and CRP have been linked to elevated endothelial microparticles in coronary heart disease [97] (Table 1).

Another acute-phase protein that serves as a biomarker of endothelial dysfunction is serum amyloid A (SAA), a family of acute-phase apolipoproteins encoded by four genes—SAA1, SAA2, SAA3, and SAA4—and produced primarily by hepatocytes and macrophages. SAA exhibits both proinflammatory and pro-atherogenic properties [1]. During the acute-phase response, circulating levels may increase by up to 1000-fold, driven by the combined stimulatory effects of TNF, IL-1, IL-6, and endogenous glucocorticoids. SAA levels are elevated in chronic vascular conditions and have been identified as predictive markers of CVD risk [98] (Table 1).

Cytokines are secreted signaling molecules that are crucial for initiating, sustaining, and resolving immune responses [99]. Proinflammatory cytokines such as TNF-α, IL-6, IL-8, and IL-18 contribute to endothelial dysfunction by upregulating adhesion molecules in ECs and leukocytes, thereby promoting leukocyte recruitment and vascular inflammation [1].

TNF-α is a major mediator of endothelial activation, inducing cytokine, chemokine, and adhesion molecule expression required for monocyte infiltration. Elevated TNF-α levels have been associated with endothelial dysfunction in hypertension, increased carotid intima-media thickness, and greater CAD risk, and have been shown to promote foam cell formation via monocyte activation [1]. Additionally, TNF-α decreases NO production by inhibiting the enzymatic activity of eNOS, while simultaneously promoting NO degradation by increasing NADPH-dependent O_2_^−^ generation, which reacts with NO to form ONOO^−^. This dual effect reduces NO bioavailability, thereby impairing smooth muscle relaxation in the vascular system [100] (Table 1).

IL-6, IL-8, and IL-18 are proinflammatory cytokines increasingly recognized for their roles in the pathogenesis of CAD. IL-6, a central mediator of the acute-phase response, has been associated with coronary plaque instability and adverse cardiovascular outcomes, in some cases demonstrating greater predictive value than high-sensitivity CRP. IL-8 functions primarily as a chemoattractant for neutrophils and T lymphocytes, with elevated levels frequently observed in CAD patients. IL-18, mainly produced by macrophages, promotes the expression of other atherogenic cytokines and has been linked to plaque destabilization and cardiovascular events. Its prognostic value has been confirmed in multiple studies and supported by meta-analyses, suggesting IL-18 as an independent marker of CAD risk [1] (Table 1). In prolonged activation, ECs can produce proinflammatory cytokines, further amplifying the recruitment of inflammatory cells. This cytokine production enhances the expression of adhesion molecules like ICAM-1 and VCAM-1, establishing an environment that supports continued immune cell activation and infiltration, ultimately resulting in chronic inflammation of the vessel wall [26].

Fibrinogen, synthetized primarily in the liver, plays a multifaceted role in the body as it possesses diverse biochemical and structural properties that enable it to engage with cells, modulate inflammatory processes, and contribute to clot formation and tissue repair, making them integral to both normal physiology and various disease mechanisms [101] (Table 1). Notably, elevated fibrinogen levels have been associated with reduced endothelial-dependent vasodilation and increased arterial stiffness, particularly in response to acute inflammation or mental stress. These responses are thought to reflect endothelial activation and reduced NO availability, linking fibrinogen to impaired vascular function and heightened cardiovascular risk. Individuals showing heightened fibrinogen responses to acute mental stress have also demonstrated increased arterial stiffness and elevated systolic blood pressure over time, suggesting a potential link between fibrinogen-mediated endothelial dysfunction and long-term cardiovascular risk [102]. There was a study in the form of a systematic review and meta-analysis conducted to clarify the association between elevated fibrinogen levels and adverse outcomes in patients with CAD. This analysis included data from 13 observational studies, comprising a total of 20,395 CAD patients. The findings revealed that patients with the highest fibrinogen levels had a significantly greater risk of cardiovascular death (risk ratio (RR) 2.24; 95% confidence interval (CI) 1.69–2.98), all-cause mortality (RR 1.88; 95% CI 1.50–2.36), and major adverse cardiovascular events (RR 1.46; 95% CI 1.18–1.81) compared to those with the lowest levels. These results suggest that baseline fibrinogen concentration is a strong and independent predictor of mortality and adverse cardiovascular outcomes in CAD, highlighting its potential utility as a biomarker for risk stratification in clinical practice [103] (Table 1).

**Table 1:** Biomarkers of endothelial dysfunction—summary.

Category	Biomarker	Source/Nature	Mechanism/Role	Clinical Relevance
Cell Adhesion Molecules	ICAM-1	Endothelial cells [94]	Mediates leukocyte adhesion/transmigration via β2 integrins; upregulated by TNF-α, IL-1β, IL-6, IFN-γ, oxidative stress [94]	Marker of vascular inflammation, CAD risk [94]
	VCAM-1	Endothelial cells [95]	Binds leukocyte integrins; promotes transendothelial leukocyte migration; induced by TNF-α, ROS, oxLDL, shear stress [95]	Associated with vascular injury, immune cell recruitment [95]
	Soluble CAMs	Circulating forms [96]	Surrogate marker of endothelial activation [90]	Measurable in plasma/serum [96]
Acute Phase Proteins	CRP	Hepatocytes [1]	Reduces eNOS expression, NO bioavailability [1]	Predictor of endothelial dysfunction, CAD, mortality [1,97]
	SAA	Hepatocytes, macrophages [1]	Proinflammatory, pro-atherogenic [1]	CVD risk prediction [1,98]
	Fibrinogen	Hepatocytes [101]	Promotes clot formation and modulates inflammation; elevated levels reduce NO-mediated vasodilation and increase arterial stiffness, especially during inflammation or stress [102,103]	Strong independent predictor of mortality and adverse CV outcomes in CAD [103]
Cytokines	TNF-α	Immune cells [1]	Induces CAM and cytokine expression; decreases NO by inhibiting eNOS and increasing ROS, leading to peroxynitrite formation and impaired vasodilation [86]	Linked to endothelial dysfunction in hypertension, ↑ carotid IMT, ↑ CAD risk, and foam cell formation [1,100]
	IL-6	Immune cells [1]	Acute phase mediator, plaque instability [1]	Predicts CAD events [1]
	IL-8	Immune cells [1]	Chemoattractant for neutrophils/T cells [1]	Elevated in CAD [1]
	IL-18	Macrophages [1]	Induces other cytokines, plaque destabilization [1]	Independent CAD risk marker [1]
Vasoactive/ Regularoty Molecules	ADMA	Endogenous molecule [104]	Inhibits NOS, reduces NO, promotes oxidative stress [104]	Predictor of CAD severity, vascular remodeling [1,104,105]
	vWF	Endothelial cells, megakaryocytes [106]	Platelet adhesion, Factor VIII stabilization [107]	Marker of vascular injury, atherosclerosis [106,107,108]
	Endothelin-1	Endothelial cells, cardiomyocytes [109]	Potent vasoconstrictor, proinflammatory [109]	Predictor of mortality in AHF, CAD [109,110]
Oxidative Stress Molecules	oxLDL	Modified LDL [97]	Activates LOX-1 → CAMs ↑, inflammation [98]	CVD risk in chronic inflammation [98,111]
	MPO	Neutrophiles, monocytes [112]	Generates oxidants, reduces NO, destabilizes plaques [113]	Marker of CAD [112,113,114]

→—results in; ↑—increase.

#### 5.1.3. Vasoactive and Endothelial Regulatory Molecules

Asymmetric dimethyl arginine (ADMA) is an endogenous compound that inhibits NOS by competing with L-arginine—its natural substrate and structural analogue. Reduced NO production due to suppression of eNOS activity contributes to the imbalance of vasoactive mediators and disruption of endothelial homeostasis. ADMA is considered one of the most potent endogenous inhibitors across all NOS iso-forms and is widely recognized as a marker of endothelial dysfunction [1]. While earlier studies primarily focused on the inhibitory effects of ADMA on NOS activity and NO production, more recent research has increasingly highlighted its role in promoting oxidative stress and fibrotic processes [104] (Table 1). ADMA’s concentrations have been linked to several cardiovascular risk factors, including hypertension, obesity, elevated triglycerides, and both type 1 and type 2 DM. Individuals with impaired endothelial function, identified by reduced fractional flow reserve in at least one vessel, were found to have significantly elevated ADMA levels, indicating its value as an independent predictor of both the severity and physiological impact of coronary atherosclerosis [1] (Table 1).

Furthermore, elevated ADMA concentrations have been negatively associated with forearm blood flow and linked to an increased incidence of cardiovascular complications and mortality. The observed correlation between ADMA levels and carotid intima-media thickness also indicates a potential role in vascular remodeling, especially among individuals with hypertension. Taken together, these findings highlight ADMA as not only a marker of endothelial dysfunction but also a valuable prognostic indicator in cardiovascular pathology [105] (Table 1).

Von Willebrand factor (vWF) is a multifunctional glycoprotein essential for primary hemostasis and coagulation. It is initially synthesized in ECs and megakaryocytes, then stored in Weibel–Palade bodies within ECs and alpha-granules of megakaryocytes. vWF plays two key roles in hemostasis: (1) mediating platelet adhesion and (2) stabilizing coagulation Factor VIII. Upon endothelial damage, vWF binds to exposed collagen, allowing platelet glycoprotein Ib receptors to attach, initiating platelet activation and aggregation [106] (Table 1). Secretion of vWF can be triggered by inflammatory mediators, primarily from tissue macrophages, leading to leukocyte adhesion, transendothelial migration, and activation of proinflammatory pathways like complement and neutrophil extracellular traps. Additionally, proinflammatory molecules such as P-selectin are co-released from Weibel–Palade bodies, further amplifying the inflammatory response [107]. The clinical value of vWF as a biomarker has been debated, as its levels may be influenced by various patient-related factors, including age, body weight, diet, smoking status, ethnicity, and physical activity. Nevertheless, its potential as a relatively affordable and non-invasive diagnostic marker has driven substantial research in CVD, leading to its recognition in identifying patients at higher risk of major adverse cardiovascular events [107] (Table 1). Owing to its involvement in vascular injury and repair, vWF has also been recognized as a marker of endothelial dysfunction, with elevated plasma levels reported in conditions such as atherosclerosis, cerebrovascular disease, and pulmonary hypertension, where higher concentrations have been linked to increased risk of complications and mortality [108] (Table 1).

ET-1 is a potent vasoconstrictive peptide mainly synthesized by ECs and cardiomyocytes. Beyond its vascular actions, it contributes to cardiometabolic disease progression through proinflammatory, profibrotic, and hypertrophic effects, with a particularly important role in heart failure. Elevated circulating ET-1 levels have been consistently associated with disease severity, increased risk of mortality, and higher rates of hospitalization. Several clinical studies have further demonstrated its value as a diagnostic and short-term prognostic biomarker, showing performance comparable to established markers such as NT-proBNP, while also appearing relatively unaffected by common comorbidities [109,110] (Table 1).

#### 5.1.4. Oxidative Stress Markers

OxLDL is a modified form of LDL cholesterol that exerts significant pro-atherogenic effects by promoting endothelial cell activation and dysfunction. It is both—a marker and a mediator of oxidative stress, contributing to the initiation and progression of atherosclerotic lesions. oxLDL interacts primarily with the LOX-1, which is abundantly expressed on ECs, leading to the upregulation of adhesion molecules, increased leukocyte recruitment, and enhanced local inflammatory responses [97] (Table 1). There were a systematic review and meta-analysis that evaluated the potential of oxLDL as a biomarker and therapeutic target for CVD risk in individuals with chronic inflammatory conditions. The study aimed to clarify whether elevated oxLDL levels could reliably indicate increased cardiovascular risk in this population. The final meta-analysis included three observational studies with a combined total of 1060 participants. It revealed a significant increase in oxLDL concentrations among patients with CVD compared to those without, highlighting its potential utility in cardiovascular risk stratification. These findings support the role of oxLDL as a promising biomarker in detecting subclinical vascular pathology and adverse outcomes among patients with chronic inflammation [111] (Table 1).

Myeloperoxidase (MPO) is a heme-containing enzyme involved in innate immunity, recognized for its proinflammatory and pro-oxidative properties that contribute to plaque destabilization [112] (Table 1). It is an important factor in inflammation-driven endothelial dysfunction and the progression of atherosclerosis. When released from activated phagocytes, MPO generates reactive oxidants such as hypochlorous acid, which reduce NO bioavailability and impair vascular function. Experimental studies in murine models and MPO-deficient mice have confirmed this role, primarily through oxidative modification of soluble guanylyl cyclase. These findings also indicate that selective MPO inhibition may serve as a potential countermeasure to endothelial dysfunction in vascular inflammation [113].

Since its association with CAD was first highlighted in 2001, MPO has been studied as a circulating biomarker in several cardiovascular conditions, including acute coronary syndrome, chronic heart failure, and myocardial infarction. Elevated plasma levels are consistently observed in CAD patients, supporting its diagnostic and prognostic relevance [112,114] (Table 1). However, its broader clinical application is currently limited by challenges such as the lack of assay standardization and difficulties in demonstrating added predictive value compared with established markers such as troponins [112]. Efforts to overcome these obstacles include development of standardized assays, validation in large patient cohorts, and investigation of MPO-targeted therapies.

### 5.2. Imaging and Functional Assessment Techniques

#### 5.2.1. Flow-Mediated Dilation

FMD is a non-invasive technique for assessing endothelial function by quantifying changes in arterial diameter in response to shear stress–induced vasodilation. This response is mediated by endothelial NO release triggered by increased blood flow. The procedure involves a pneumatic cuff is inflated on the forearm to supra-systolic pressure for approximately five minutes, thereby occluding blood flow in the brachial artery. Upon cuff deflation, the resultant increase in the blood flow elevates shear stress on the arterial wall, stimulating NO production and subsequent vasodilation [115].

FMD assessment involves measuring the brachial artery diameter before cuff inflation and after cuff release, with the percentage change calculated relative to baseline. Given the small artery size (3–4 mm), even minor measurement errors can significantly affect results; therefore, advanced vessel wall detection software is often applied to improve accuracy beyond the 0.1 mm resolution of standard ultrasound devices [116]. 

Deflation of the cuff leads to a substantial increase in blood flow as distal vascular resistance decreases, whereas individuals with vascular dysfunction exhibit a reduced vasodilatory response [117].

Meta-analytic evidence indicates that each 1% increase in FMD is associated with a 13% reduction in cardiovascular event risk, and a one standard deviation increase corresponds to a 41% lower risk [116]. FMD is also influenced by cardiovascular risk factors, correlates with coronary artery endothelial function, and can be applied to study both—the acute and long-term effects of physiological or pharmacological interventions. Given its strong association with various cardiovascular risk factors and conditions, FMD offers a practical approach for early detection and monitoring of endothelial dysfunction in at-risk populations. When applied under standardized conditions, it can offer robust prognostic insights to guide prevention and management strategies in patients with or at risk for CVD [117,118]. However, FMD results remain influenced by operator skill and device variability. Standardized protocols and automated analysis are improving reproducibility and clinical reliability.

#### 5.2.2. Peripheral Arterial Tonometry & Reactive Hyperemia Index

While FMD primarily evaluates endothelial function in conduit arteries, Peripheral Arterial Tonometry (PAT) provides a complementary assessment focused on the microvasculature and peripheral arterial tone. PAT with the EndoPAT device is a non-invasive method for assessing endothelial function. It measures changes in fingertip pulse wave amplitude during a FMD procedure, where reactive hyperemia induced by temporary arterial occlusion increases shear stress on vessel walls. This shear stress triggers NO release, promoting local vasodilation. EndoPAT uses pneumatic probes on both index fingers—one arm undergoes occlusion while the other serves as a control to account for systemic autonomic influences. The device calculates the Reactive Hyperemia Index (RHI) as the ratio of post- to pre-occlusion PAT signal amplitude, normalized to the control arm, with baseline correction applied to adjust for initial signal amplitude [119]. A reduced RHI reflects impaired endothelial function, shows an association with coronary endothelial dysfunction, and serves as an important predictor of future cardiovascular events. However, its correlation with FMD—an indicator of conduit vessel NO bioavailability—is only modest [120]. RHI measurements also face device- and operator-related variability, though advances in automation and standardization are enhancing consistency and clinical applicability.

#### 5.2.3. Pulse Wave Analysis

In addition to techniques that rely on diameter changes in response to shear stress, endothelial function can also be assessed by analyzing the arterial pulse waveform, which provides insight into vascular compliance and arterial stiffness. Pulse wave analysis (PWA) is a non-invasive diagnostic technique used to characterize the hemodynamic properties of the arterial system. By examining the morphology and timing of the arterial pulse waveform, PWA yields valuable information on vascular stiffness and compliance, thereby serving as an important tool for assessing both cardiovascular health and endothelial function [121].

Several non-invasive approaches, including PAT, have been utilized to link changes in the pulse waveform with endothelial responses during reactive hyperemia. These methods show consistent associations with established ultrasound-based measurements of flow-mediated dilation, supporting their validity as practical alternatives for endothelial function testing. Key advantages of PWA-based approaches include their ease of implementation, minimal training requirements, short measurement duration for participants, non-invasive nature, portability, and overall cost-efficiency [121,122].

#### 5.2.4. Venous Occlusion Plethysmography

Whereas PWA evaluates systemic hemodynamics, venous occlusion plethysmography (VOP) focuses on localized forearm blood flow and resistance, offering another view of endothelial function. It works by partially restricting venous outflow while maintaining arterial inflow—typically achieved by inflating an upper-arm cuff to around 40 mm Hg. To exclude the hand from circulation, a second cuff is placed at the wrist and inflated to suprasystolic pressure (approximately 200 mm Hg). Under these conditions, the forearm gradually swells as blood enters but cannot exit through the veins. The rate and extent of this swelling, measured with a voltage-dependent strain gauge, are directly proportional to blood flow and allow calculation of vascular resistance. A modified, minimally invasive strain-gauge VOP method can be used to assess endothelial function in vivo, making it a useful research tool in cardiovascular physiology [123]. This method is recognized for evaluating vascular function, including endothelium-dependent vasodilation. Studies using this technique have shown that patients with chronic heart failure and systolic dysfunction exhibit significantly reduced vasodilatory capacity compared to healthy controls. This impairment likely stems from an imbalance between NO availability and oxidative stress, contributing to reduced myocardial perfusion and potentially driving the progression of heart failure [124].

#### 5.2.5. Intracoronary Acetylcholine Provocation Test

The intracoronary acetylcholine (ACh) provocation test is an invasive diagnostic method for assessing coronary endothelial function. It is recommended in contemporary clinical guidelines and is widely used in practice, demonstrating a favorable safety profile when performed by experienced operators [125]. 

Acetylcholine, a neurotransmitter of the parasympathetic nervous system, exerts its effects through both nicotinic and muscarinic (mAChR) receptors. The latter play a pivotal role in vascular homeostasis, with ACh acting as a non-selective agonist. Stimulation of endothelial mAChRs induces NO–mediated vasodilation, whereas activation of mAChRs located on vascular smooth muscle cells results in vasoconstriction. Consequently, the net vascular response to intracoronary administration of ACh—vasodilation or vasoconstriction—depends on the integrity of the endothelium and the contractile reactivity of smooth muscle cells [125].

Vasoreactivity testing with ACh serves two principal diagnostic purposes: evaluation of coronary endothelial function and detection of coronary microvascular or epicardial spasm. However, protocols for ACh administration vary among clinicians, with modifications used to diagnose epicardial spasm, microvascular spasm, or endothelial dysfunction. Standardization of ACh testing procedures for the assessment of both coronary spasm and endothelial function remains a critical objective for cardiology practice [126].

#### 5.2.6. Thermodilution or Doppler Flow Wire Measurements

Coronary Flow Reserve (CFR) represents the ratio between maximal achievable coronary blood flow (during hyperaemia) and resting flow, reflecting the combined function of epicardial arteries and the coronary microcirculation. A reduced CFR may indicate epicardial stenosis, microvascular dysfunction, or both [127]. Thermodilution is a pressure–temperature sensor–based technique used to quantify CFR and the Index of Microcirculatory Resistance (IMR). It involves rapid intracoronary injections of room-temperature saline, measuring the resultant temperature changes to calculate mean transit time of blood at rest and during hyperaemia [128]. In the context of endothelial dysfunction, impaired microvascular dilatory capacity—often due to reduced NO bioavailability and increased vasoconstrictor tone—leads to a diminished CFR, even in the absence of significant epicardial stenosis. Thus, CFR and thermodilution-derived IMR provide valuable physiological markers of microvascular and endothelial health [129].

## 6. Targeted Therapies for Endothelial Dysfunction

### 6.1. Pharmacological Approaches

Endothelial dysfunction, characterized by impaired NO bioavailability, increased oxidative stress, and a proinflammatory vascular phenotype, plays a central role in the pathogenesis of atherosclerosis, diabetes-related vascular complications, and other cardiovascular disorders [5]. Pharmacological interventions that target eNOS activity, NO bioavailability, and vascular inflammation represent important strategies for restoring endothelial homeostasis. Angiotensin-converting enzyme (ACE) inhibitors exert vasculoprotective effects that extend beyond blood pressure reduction. By blocking the conversion of angiotensin I to angiotensin II, they decrease vasoconstriction, oxidative stress, and inflammation while enhancing bradykinin-mediated eNOS activation. Acute administration of ACE inhibitors has been shown to improve endothelium-dependent vasodilation in human coronary arterioles, an effect mediated by increased NO production and reduced superoxide generation [130].

Statins, although widely recognized for their lipid-lowering properties, also possess pleiotropic effects that directly modulate endothelial function. They inhibit RhoA/Rho kinase signaling, which stabilizes eNOS mRNA and promotes eNOS phosphorylation, thereby enhancing NO bioavailability. Improvements in endothelial vasodilatory responses in the coronary microcirculation have been observed following statin therapy, with these effects being independent of lipid lowering and partly attributable to reductions in oxidative stress and vascular inflammation [5,130]. Glitazones, as agonists of peroxisome proliferator-activated receptor gamma (PPARγ), exhibit insulin-sensitizing and anti-inflammatory actions. They can improve endothelial function in diabetic patients by reducing oxidative stress markers and restoring eNOS coupling [131]. PPARγ activation modulates transcription of antioxidant genes, decreases the expression of vascular cell adhesion molecules, and suppresses proinflammatory cytokine release, ultimately enhancing NO-mediated vasodilation. These effects are particularly important in DM, where hyperglycemia-driven oxidative stress is a major cause of endothelial dysfunction [132].

Metformin, the first-line treatment for T2DM, confers vascular benefits that extend beyond glycemic control. It activates the AMP-activated protein kinase (AMPK) pathway, leading to eNOS phosphorylation, increased NO production, and improved endothelium-dependent vasodilation [133]. Metformin reduces mitochondrial ROS generation, preventing NO degradation. It also downregulates NF-κB signaling and suppresses vascular adhesion molecule expression. Collectively, these mechanisms enhance endothelial homeostasis and may contribute to cardiovascular protection in both diabetic and non-diabetic populations [133].

Recent evidence suggests that metformin and other metabolic interventions can modulate fibroblast growth factor 21 (FGF21) signaling, which is implicated in energy homeostasis, vascular inflammation, and oxidative stress regulation [134]. Activation of the AMPK–ATF4 axis by metformin may upregulate FGF21 expression, providing an additional mechanism for improving NO bioavailability and endothelial function. While the clinical implications of FGF21 modulation for vascular health remain under investigation, this represents a promising avenue for future pharmacological approaches [134].

Agonists of the glucagon-like peptide-1 receptor (GLP-1 RAs) mimic the effects of endogenous GLP-1, a hormone involved in glucose homeostasis, insulin secretion, appetite regulation, and cardiovascular function. These agents enhance insulin release, suppress glucagon secretion, slow gastric emptying, and improve endothelial function. Regarding the effects of GLP-1 receptor agonists on the vascular endothelium, both direct actions on endothelial cells and effects on vascular smooth muscle cells have been reported. Significant reductions in major adverse cardiovascular events (MACE) have also been documented in large-scale clinical trials. Please include this information as well [135].

ACE inhibitors, statins, glitazones, and metformin act through different but converging molecular pathways that influence eNOS activity, NO bioavailability, and vascular inflammation. By reducing oxidative stress, suppressing inflammatory signaling, and promoting NO-mediated vasodilation, these agents not only acutely improve endothelial function but may also provide long-term cardiovascular protection. Combination therapies employing complementary mechanisms—for example, pairing statins with ACE inhibitors—may yield additive or synergistic benefits, as demonstrated in microvascular studies [130]. Given the central role of endothelial dysfunction in CVD pathophysiology, optimizing pharmacological strategies to enhance eNOS function and NO bioavailability remains a pivotal objective in preventive and therapeutic cardiometabolic medicine.

### 6.2. Non-Pharmacological Intervention

Non-pharmacological interventions are foundational in managing endothelial dysfunction and low-grade inflammation, both key drivers of cardiovascular and metabolic diseases. Among these approaches, adherence to the Mediterranean dietary pattern has been extensively studied and consistently shown to exert beneficial effects on vascular health. Comprehensive systematic reviews and meta-analyses of interventional studies demonstrate that the Mediterranean diet significantly reduces inflammatory markers and enhances endothelial function. These effects are largely attributed to the diet’s rich content of antioxidants, polyphenols, monounsaturated fatty acids, particularly from olive oil, and omega-3 fatty acids found in fish. The Mediterranean diet’s characteristic high intake of fruits, vegetables, whole grains, legumes, nuts, and olive oil, combined with moderate consumption of fish and red wine and limited intake of red meat and processed foods, collectively contribute to increased eNOS activity and NO bioavailability, thereby improving vascular dilation and exerting anti-inflammatory effects [136]. Clinical investigations involving patients with CAD corroborate the positive impact of the Mediterranean diet on endothelial function indices, such as FMD, along with a reduction in circulating inflammatory biomarkers. These beneficial outcomes are reflective of enhanced eNOS expression and NO production, which together promote vascular health and mitigate inflammatory processes. Furthermore, the diet’s positive influence extends to improvements in erectile function among individuals with metabolic syndrome, underscoring its systemic vascular benefits [137,138]. Weight loss achieved through dietary interventions, including very-low-carbohydrate diets, has been shown to improve endothelial function in overweight individuals, as measured by flow-mediated dilation (FMD). Both low- and high-carbohydrate diets improved endothelial function and cardiovascular risk markers in individuals with abdominal obesit [139].

Sex hormones and aging significantly modulate endothelial function. Estrogen in premenopausal women enhances NO production and maintains vascular health, whereas postmenopausal estrogen decline increases the risk of endothelial dysfunction. Aging is also associated with reduced eNOS activity and elevated oxidative stress, contributing to impaired endothelial function.

Physical activity levels and sedentary behavior are strongly linked with biomarkers indicative of endothelial dysfunction and chronic low-grade inflammation in populations with metabolic impairments. Elevated physical activity promotes upregulation of eNOS and augmented NO synthesis, contributing to improved endothelial health, whereas prolonged sedentary time correlates with deterioration of these parameters. Regular aerobic exercise has been shown to enhance eNOS activity, increase NO bioavailability, reduce oxidative stress, and improve endothelial function, as measured by FMD. Different exercise modalities, including moderate-intensity continuous training and high-intensity interval training, exert beneficial effects on vascular function and systemic inflammatory markers. Combined lifestyle interventions including diet, physical activity, and weight management synergistically promote endothelial health by enhancing NO production, reducing oxidative stress, and modulating inflammatory pathways. These findings emphasize the crucial role of lifestyle in maintaining vascular homeostasis and modulating inflammatory pathways [140]. NO synthases, particularly eNOS, undergo tight regulatory control and play a central role in maintaining vascular function and suppressing inflammatory responses [4].

Emerging research on fasting-mimicking dietary regimens has revealed potential to stimulate β-cell regeneration via Ngn3-dependent pathways, offering promise in reversing diabetic pathology. Although direct effects on endothelial function require further elucidation, improvements in glucose metabolism and reductions in systemic inflammation through these dietary interventions may indirectly enhance vascular health [141].

### 6.3. Emerging Targeted Therapies

Recent advances have shifted the therapeutic paradigm for endothelial dysfunction toward precision interventions that specifically target the inflammatory pathways underlying vascular injury. Cytokine-directed biologics, such as monoclonal antibodies against interleukin-1β (IL-1β), exemplify this approach by directly modulating proinflammatory signaling cascades. IL-1β plays a central role in the sterile inflammatory axis, driving the NLRP3–IL-1β–IL-6–CRP pathway, which promotes vascular inflammation and endothelial activation.

Targeted inhibition of IL-1β with canakinumab has been shown to reduce systemic inflammatory markers, such as hsCRP, and to decrease recurrent major cardiovascular events in post–myocardial infarction patients with persistent inflammation, without affecting lipid levels [142]. These findings suggest that selective anti-inflammatory therapy may indirectly support endothelial health by mitigating inflammation-induced vascular injury. However, the use of IL-1β inhibitors is associated with several limitations. Their use is limited by cost, infection risk, and the need for precise patient selection. Canakinumab, for example, has been associated with an increased risk of serious infections, including tuberculosis and fungal infections, necessitating careful screening and monitoring of patients. Additionally, the high cost of these biologic agents may limit their accessibility and widespread use, particularly in resource-limited settings [143].

Similarly, IL-17A blockade, initially developed for immune-mediated disorders such as psoriasis, has demonstrated vascular benefits, including attenuation of arterial inflammation and improvement of endothelial function in patients with chronic immune activation [144]. However, their use is limited by cost, infection risk, and the need for precise patient selection. The administration of IL-17A inhibitors has been associated with an increased risk of infections, including upper respiratory tract infections and fungal infections, which may pose significant concerns in patients with compromised immune systems. Furthermore, the high cost of these therapies may restrict their availability and use in clinical practice [145].

Another emerging avenue involves inhibition of adhesion molecules, particularly P-selectin, which mediates leukocyte and platelet adhesion to activateendothelium during ischemia–reperfusion injury. P-selectin blockade with inclacumab reduces leukocyte infiltration and microvascular obstruction, protecting the endothelium. By decreasing endothelial inflammation and oxidative stress, P-selectin antagonism helps preserve endothelial nitric oxide synthase (eNOS) function and nitric oxide (NO) bioavailability, key mediators of vasodilation and vascular homeostasis. Clinical studies have shown that pre-procedural administration of P-selectin antagonists before percutaneous coronary intervention significantly decreases periprocedural myocardial injury, as reflected by reduced troponin and CK-MB release, with the strongest effects observed when therapy is given shortly before reperfusion [146].

Chemokine-axis inhibitors, targeting pathways such as CCL2–CCR2, represent another precision-based strategy to preserve endothelial function. By preventing monocyte recruitment into the vascular wall, these agents interrupt a central mechanism driving local inflammation and oxidative stress, both of which contribute to endothelial nitric oxide synthase (eNOS) uncoupling and reduced nitric oxide (NO) bioavailability. The resulting protection of eNOS activity helps maintain endothelium-dependent vasodilation and vascular homeostasis. Preclinical models have consistently demonstrated that chemokine blockade reduces atherogenesis and vascular inflammation, effects that are closely linked to improved endothelial performance. Nevertheless, pharmacological inhibition of CCR2 has been associated with potential adverse effects, including neurotoxicity and impaired cognitive function, indicating that careful evaluation of safety and off-target consequences is essential in translational and clinical applications [147].

Beyond targeting inflammation and adhesion, metabolic modulators such as AdipoRon offer additional pathways to enhance endothelial function. AdipoRon, a synthetic agonist of adiponectin receptors AdipoR1 and AdipoR2, shows promising effects in improving endothelial function, particularly in the context of metabolic and cardiovascular diseases. Recent studies provide new evidence on the mechanisms through which AdipoRon may beneficially influence vascular health.

In studies conducted on human endothelial cells (HUVECs), AdipoRon was shown to counteract metabolic disturbances induced by TNF-α, including increased glucose metabolism and alterations in lipid metabolism and mitochondrial function. Additionally, AdipoRon modulates the lipid profile of endothelial cells by reducing ceramide levels and regulating S1P levels, which may contribute to endothelial stability and its protective function [148]. Furthermore, studies in type 2 diabetic mice suggest that AdipoRon improves endothelial function in mesenteric arteries, which may contribute to the overall improvement of vascular function in diabetic patients [149]. In the context of sepsis, AdipoRon has been shown to increase nitric oxide (NO) production and reduce oxidative stress, helping maintain vascular integrity and prevent complications associated with endothelial dysfunction [52]. These findings indicate the potential of AdipoRon as a therapeutic agent to enhance endothelial function, especially in metabolic diseases and inflammatory states. However, before its broader clinical application, further studies are needed to evaluate safety, pharmacokinetics, and efficacy in human populations.

## 7. Conclusions

Endothelial dysfunction represents an early and pivotal event in the development of cardiometabolic diseases, serving as both a marker and a driver of disease progression. It arises from the complex interplay of oxidative stress, chronic inflammation, adipokine imbalance, and reduced nitric oxide (NO) bioavailability. These mechanisms compromise vascular tone, increase leukocyte adhesion, and promote structural vessel wall changes, laying the groundwork for atherosclerosis, hypertension, diabetic vascular complications, and obesity-related cardiovascular risk.

The identification of biomarkers—including adhesion molecules (ICAM-1, VCAM-1), acute-phase proteins (CRP, fibrinogen, SAA), cytokines (TNF-α, IL-6, IL-18), vasoactive mediators (ADMA, endothelin-1, vWF), and oxidative stress indicators (oxLDL, MPO)—provides valuable tools for early detection and prognosis. Coupled with functional assessments such as flow-mediated dilation (FMD), peripheral arterial tonometry, pulse wave analysis, and intracoronary acetylcholine testing, these measures enable the more precise characterization of endothelial status and cardiovascular risk.

Targeted therapies can address multiple pathogenic pathways. Pharmacological interventions—such as ACE inhibitors, statins, glitazones, and metformin—improve endothelial function by enhancing eNOS activity, restoring NO bioavailability, reducing oxidative stress, and suppressing proinflammatory signaling. These effects are often complementary, and combined regimens may yield synergistic benefits. Equally important are non-pharmacological strategies, with strong evidence supporting the Mediterranean diet, physical activity, and weight reduction in improving endothelial health. Rich in antioxidants, polyphenols, and omega-3 fatty acids, the Mediterranean dietary pattern enhances eNOS expression, increases NO production, and lowers systemic inflammation.

Given the central role of endothelial dysfunction in linking metabolic disorders with cardiovascular disease, integrating biomarker-driven diagnostics with multimodal therapeutic approaches offers the best opportunity to prevent disease progression. Early, targeted, and sustained intervention—addressing both vascular biology and lifestyle factors—can improve vascular resilience, reduce cardiometabolic risk, and ultimately enhance patient outcomes.

## Data Availability

No new data were created or analyzed in this study. Data sharing is not applicable to this article.

## Abbreviations

The following abbreviations are used in this manuscript:

ACh	Acetylcholine
ACE	Angiotensin-Converting Enzyme
ADMA	Asymmetric Dimethylarginine
AGEs	Advanced Glycation End Products
AHF	Acute Heart Failure
AMPK	AMP-Activated Protein Kinase
ASCVD	Atherosclerotic Cardiovascular Disease
BAUS	British Association of Urological Surgeons
BH_2_	Dihydrobiopterin
BH_4_	Tetrahydrobiopterin
CAD	Coronary Artery Disease
CAMs	Cell Adhesion Molecules
CFR	Coronary Flow Reserve
cGMP	Cyclic Guanosine Monophosphate
CI	Confidence Interval
CRP	C-Reactive Protein
CVD	Cardiovascular Disease
CVS	Cardiovascular System
DM	Diabetes Mellitus
ECs	Endothelial Cells
EDCFs	Endothelium-Derived Contracting Factors
EDHF	Endothelium-Derived Hyperpolarizing Factor in Vascular
EDRFs	Endothelium-Derived Relaxing Factors
eNOS	Endothelial Nitric Oxide Synthase
ET	Endothelin
ET-1	Endothelin-1
FID	Flow-Induced Dilation
FMD	Flow-Mediated Dilation
FGF21	Fibroblast Growth Factor 21
HDL	High-Density Lipoprotein
H_2_O_2_	Hydrogen Peroxide
ICAM-1	Intercellular Adhesion Molecule-1
IL-1β	Interleukin-1 Beta
IL-17A	Interleukin-17A
IL-6	Interleukin-6
JAK/STAT	Janus Kinase/Signal Transducer and Activator of Transcription Pathway
LDL	Low-Density Lipoprotein
LOX-1	Lectin-Like Oxidized LDL Receptor-1
MCP-1	Monocyte Chemoattractant Protein-1
mAChR	Muscarinic Acetylcholine Receptor
MPO	Myeloperoxidase
NF-κB	Nuclear Factor Kappa-Light-Chain-Enhancer of Activated B Cells
NO	Nitric Oxide
NOS	Nitric Oxide Synthase
NOX2	NADPH Oxidase 2
O_2_^−^	Superoxide Anion
ONOO^−^	Peroxynitrite
oxLDL	Oxidized Low-Density Lipoprotein
PAF	Platelet-Activating Factor
PAI-1	Plasminogen Activator Inhibitor-1
PAT	Peripheral Arterial Tonometry
PGH_2_	Prostaglandin H_2_
PGI_2_	Prostacyclin
PI3K	Phosphoinositide 3-Kinase
PKC	Protein Kinase C
PKG	Protein Kinase G
PPARγ	Peroxisome Proliferator-Activated Receptor Gamma
PVAT	Perivascular Adipose Tissue
PWA	Pulse Wave Analysis
RHI	Reactive Hyperemia Index
ROS	Reactive Oxygen Species
RR	Relative Risk
SAA	Serum Amyloid A
sCAMs	Soluble Cell Adhesion Molecules
sGC	Soluble Guanylyl Cyclase
SMCs	Smooth Muscle Cells
SR-A	Scavenger Receptor Class A
T2DM	Type 2 Diabetes Mellitus
TNF-α	Tumor Necrosis Factor-Alpha
VCAM-1	Vascular Cell Adhesion Molecule-1
vWF	von Willebrand Factor
VOP	Vascular Oscillatory Pressure
